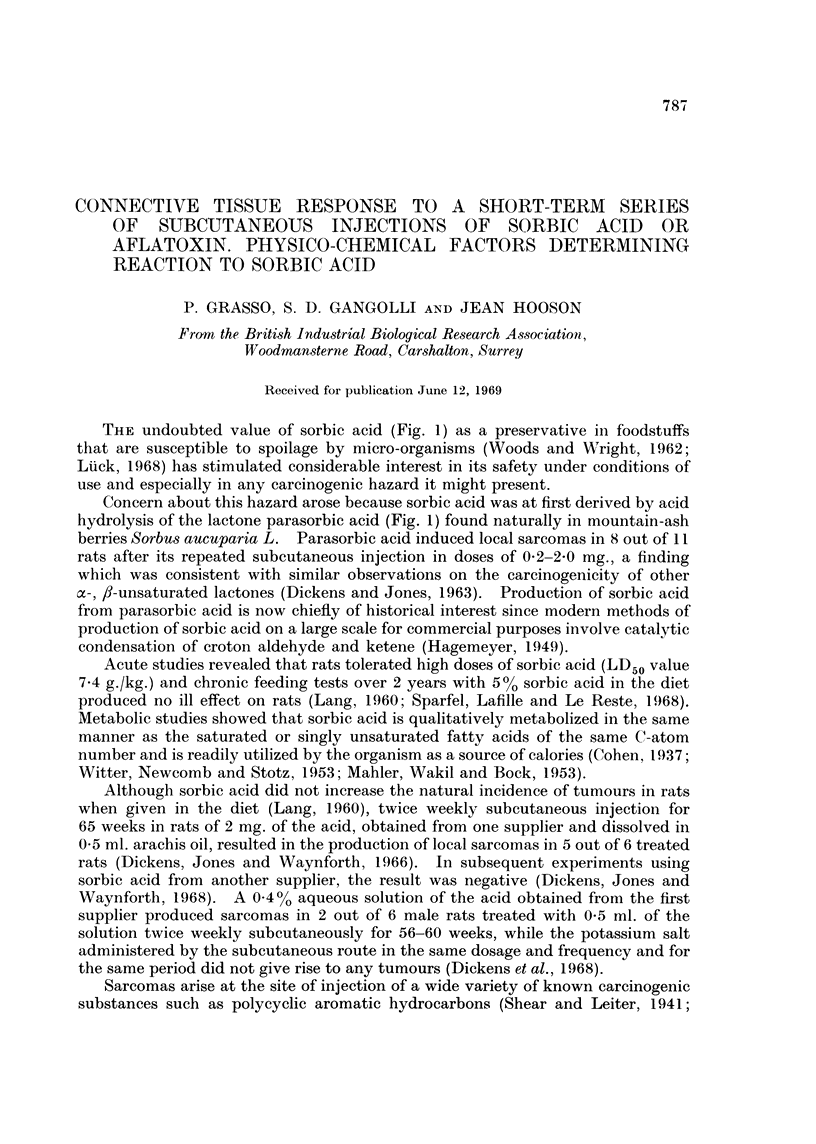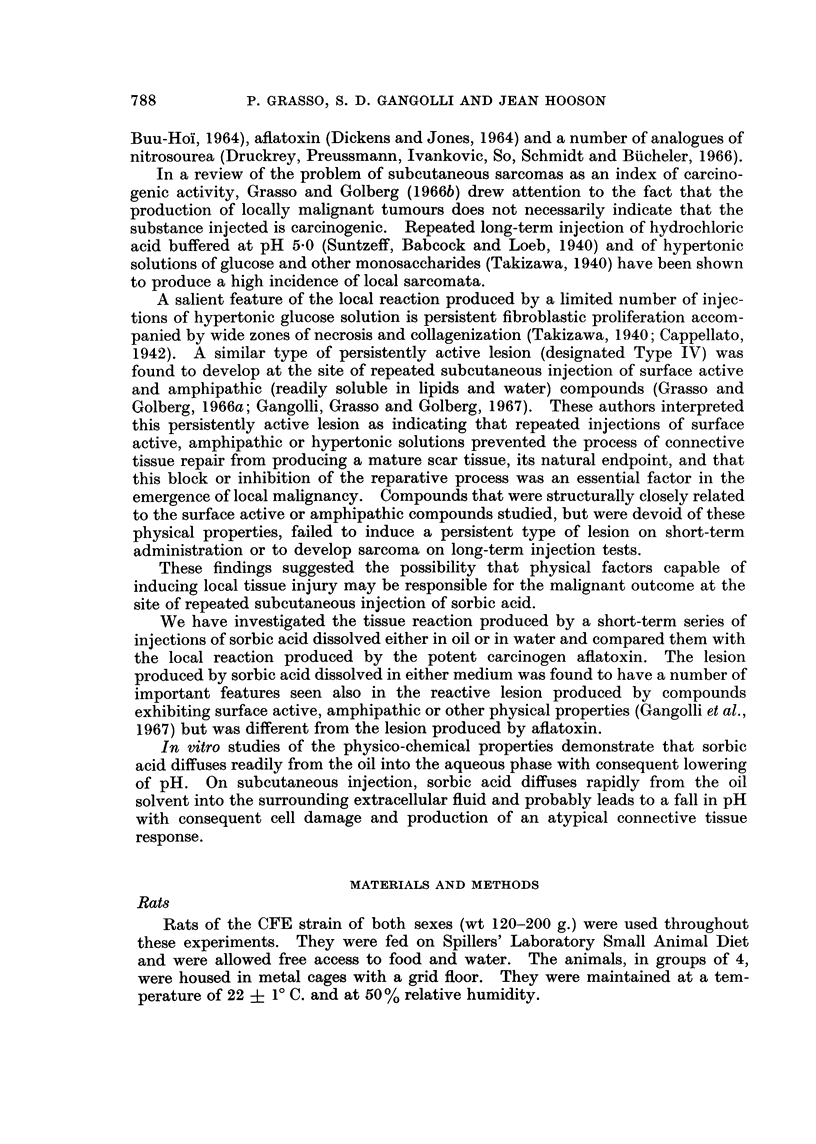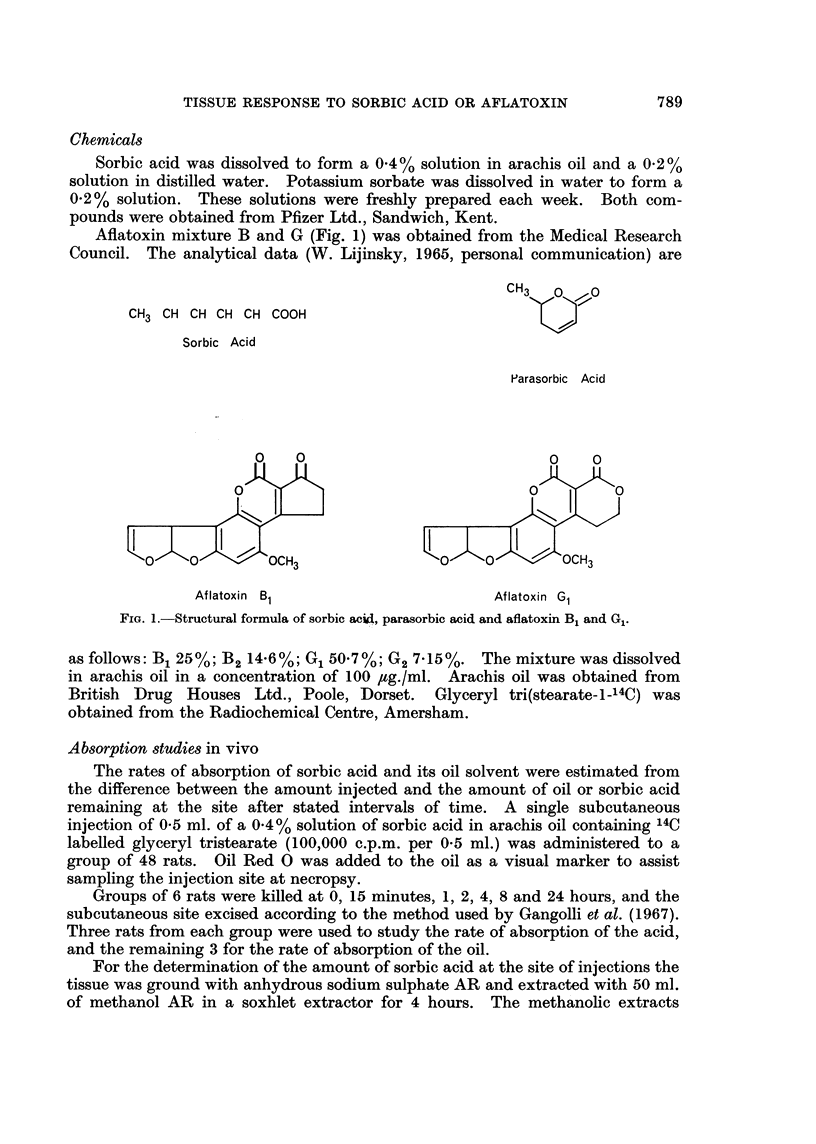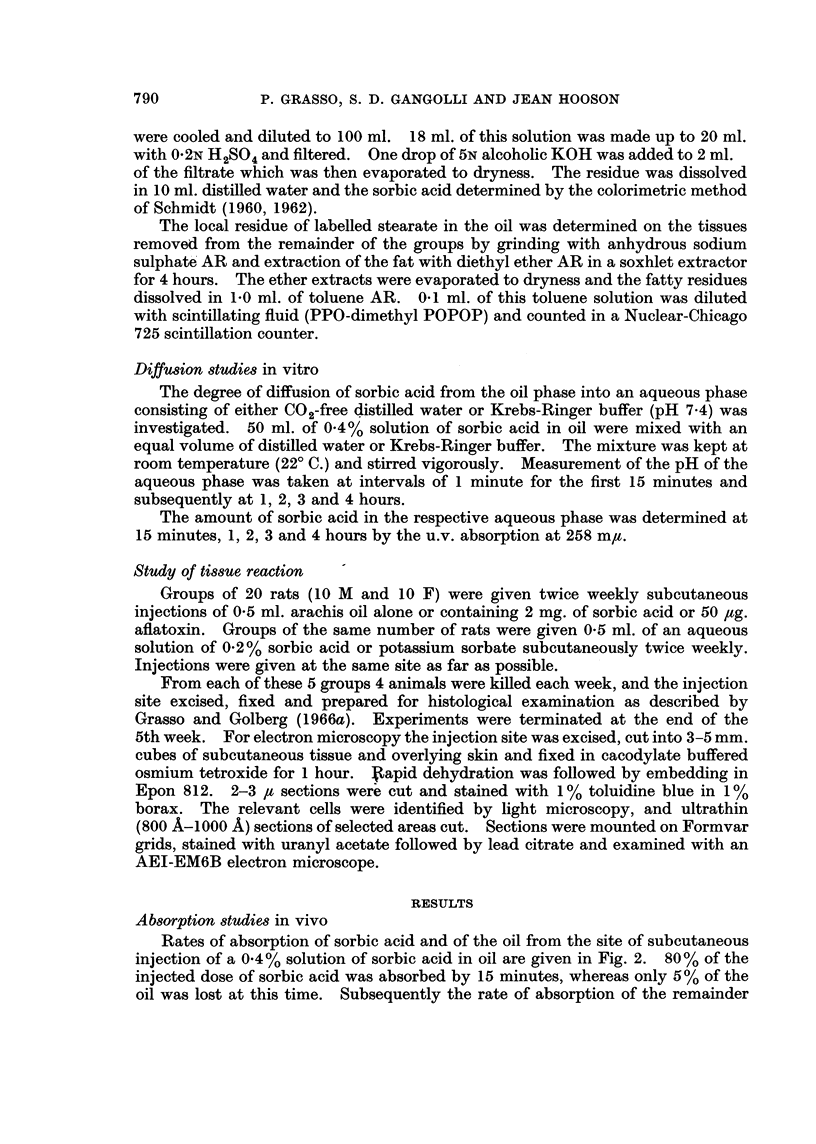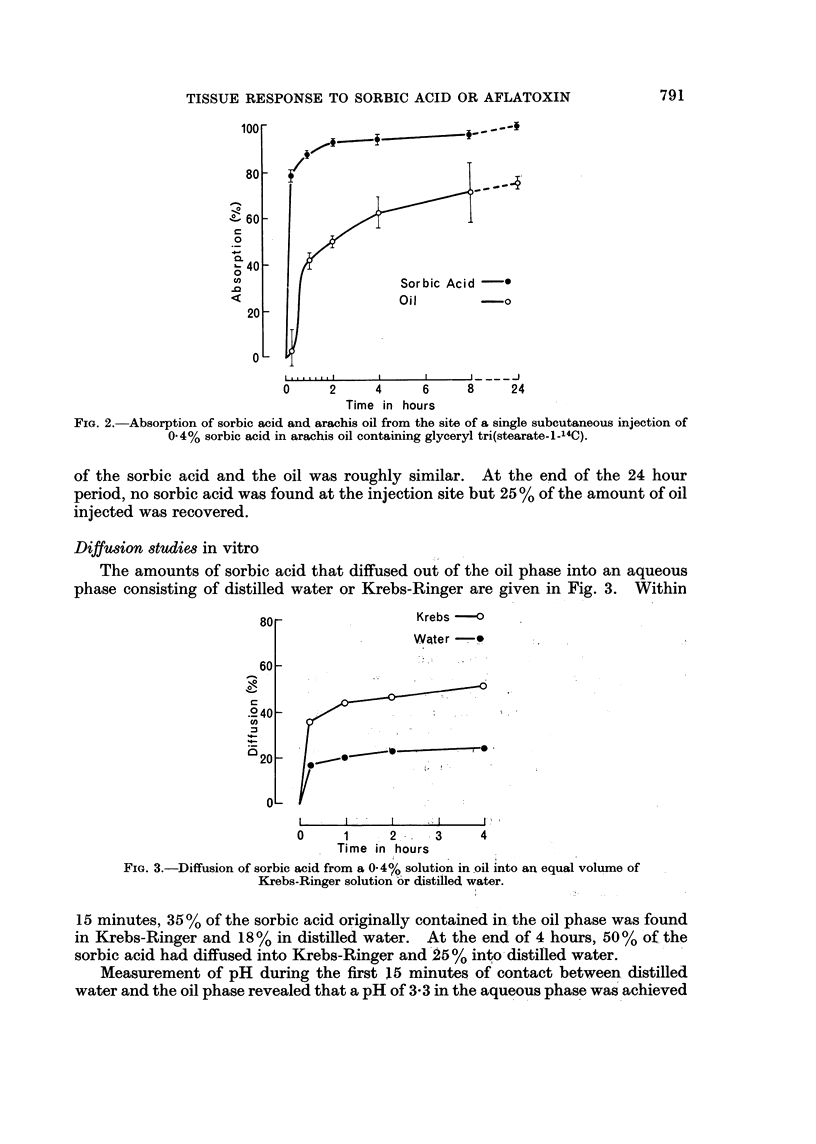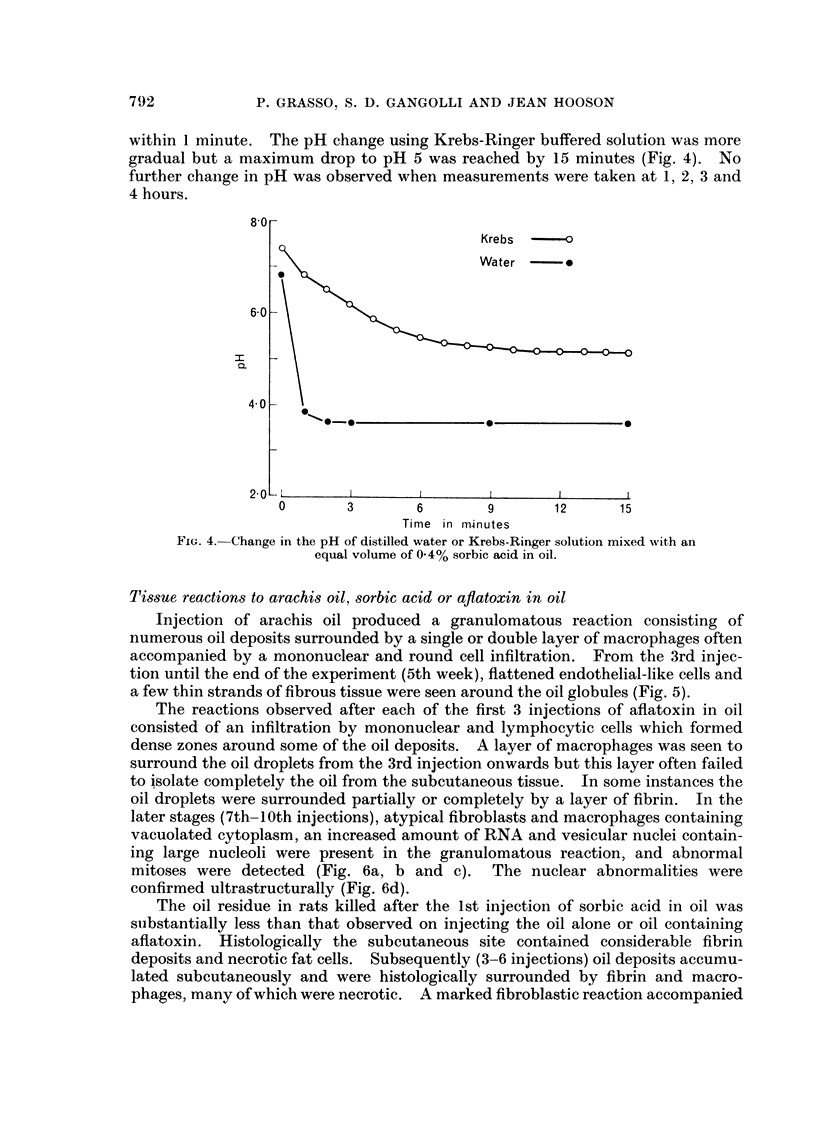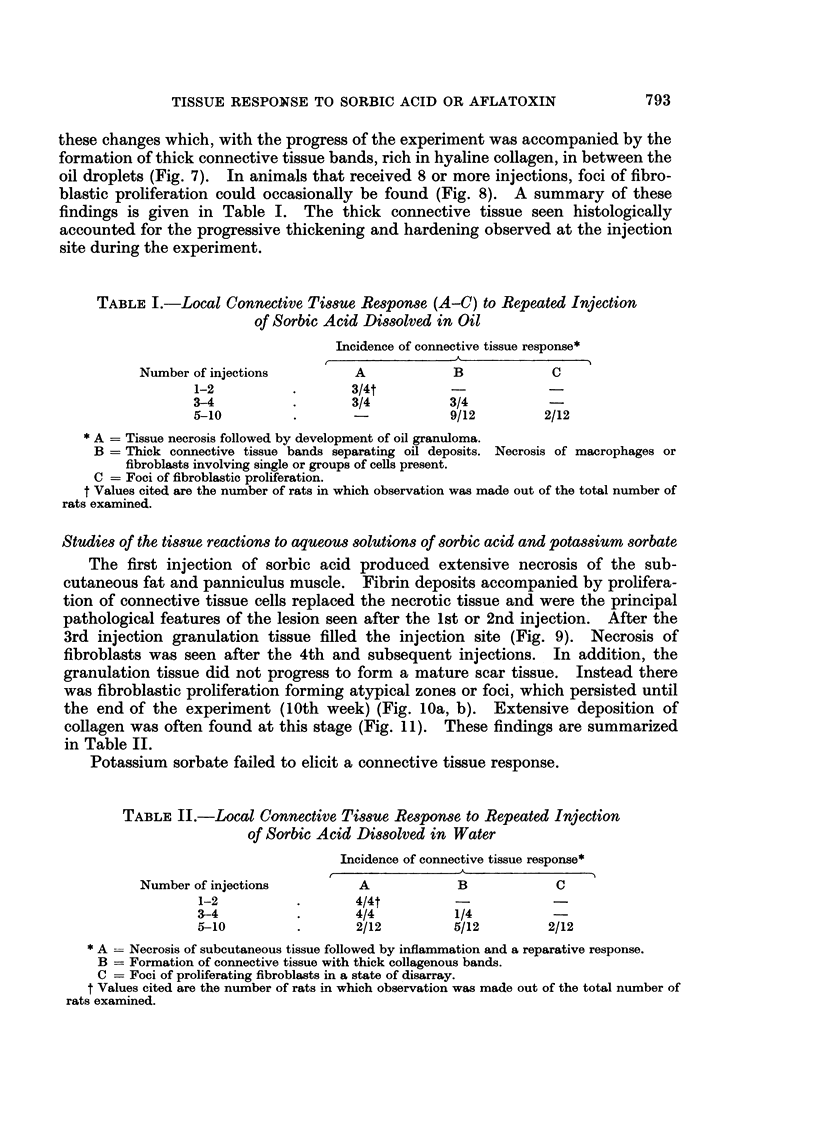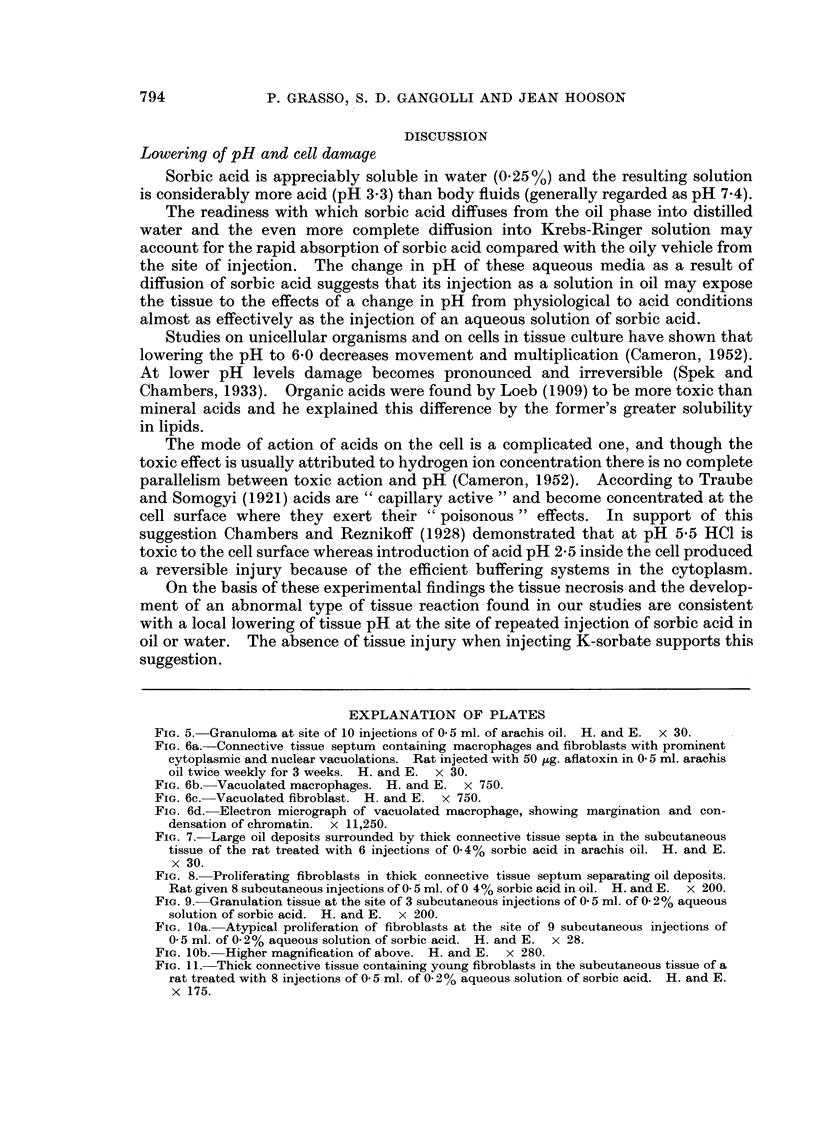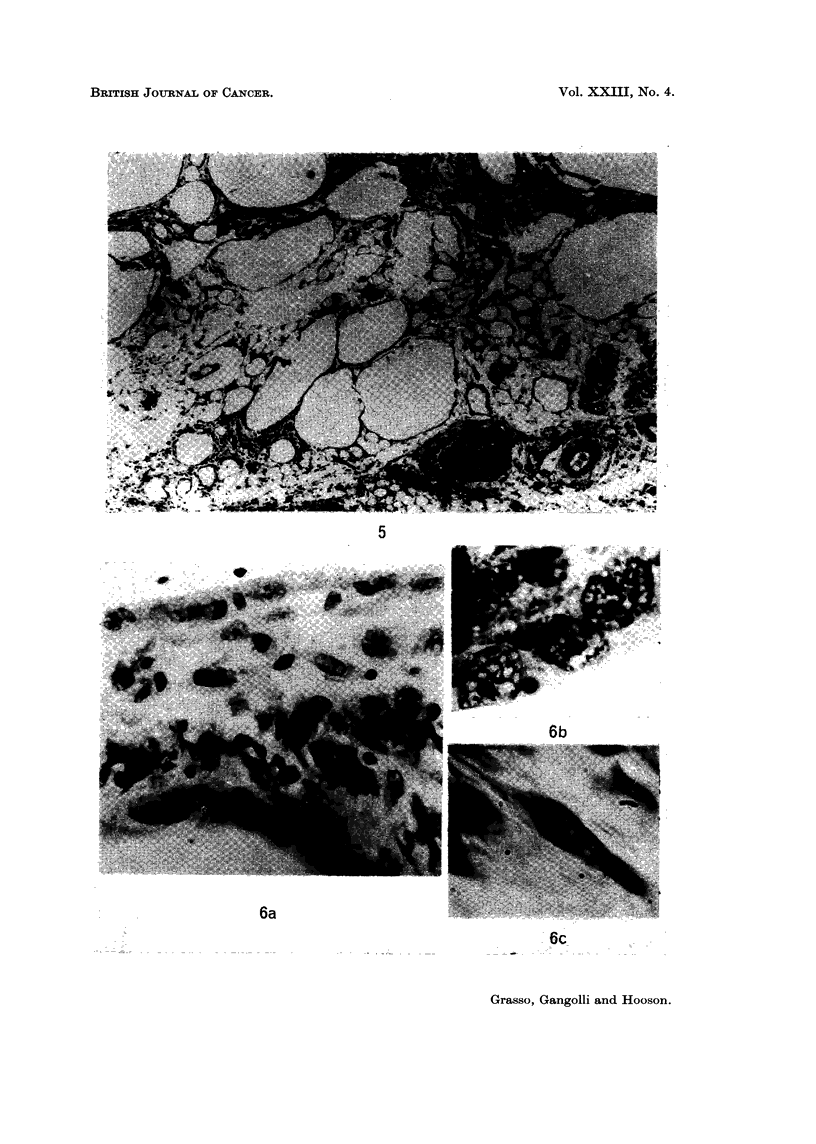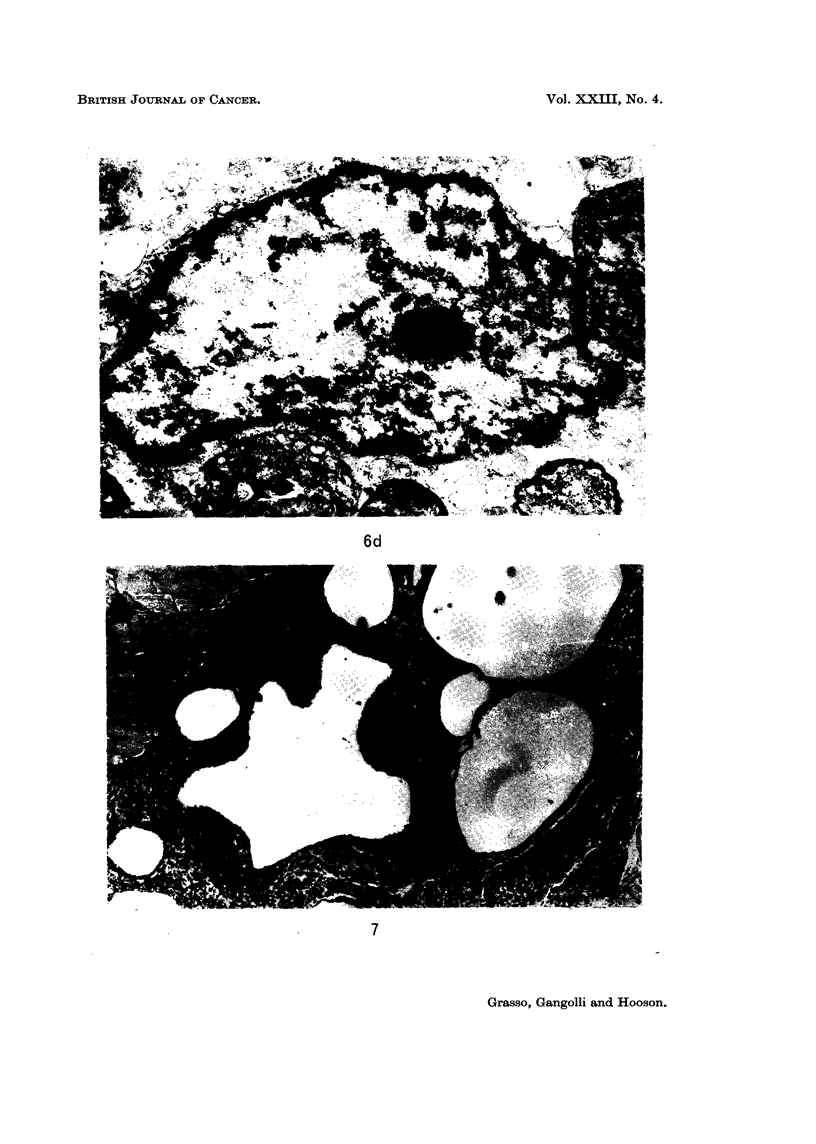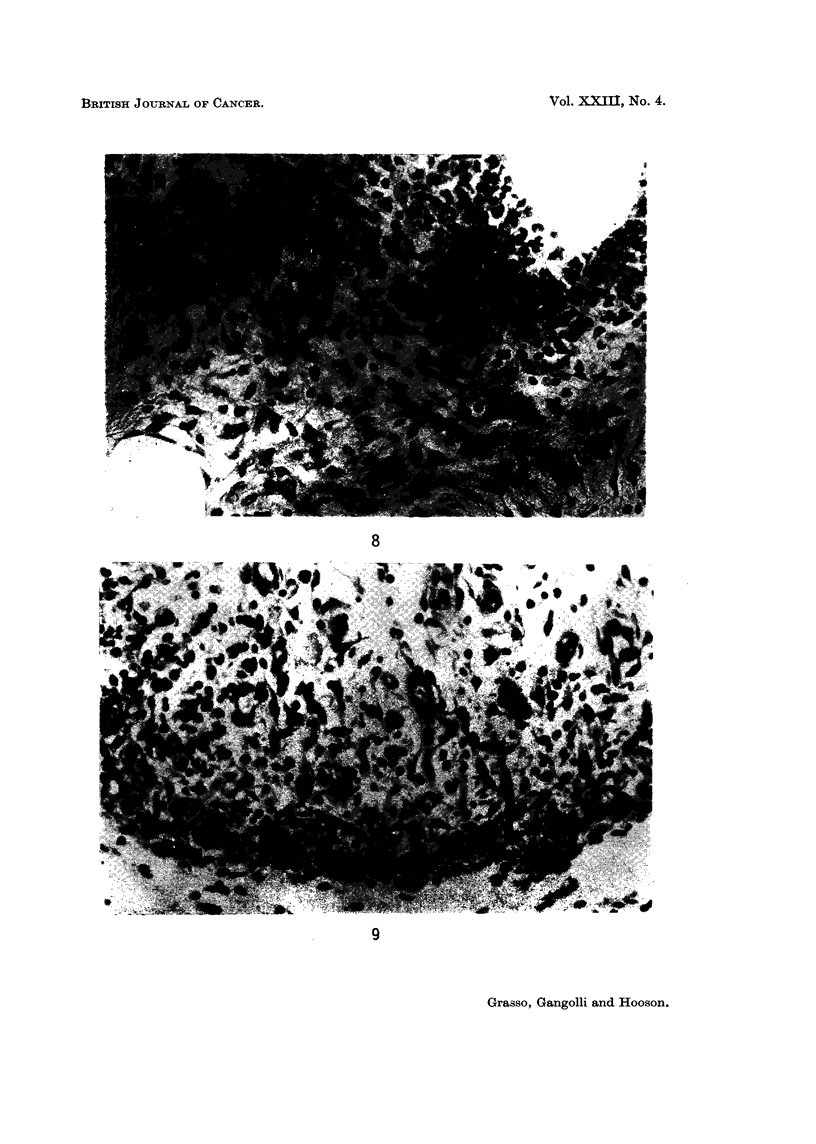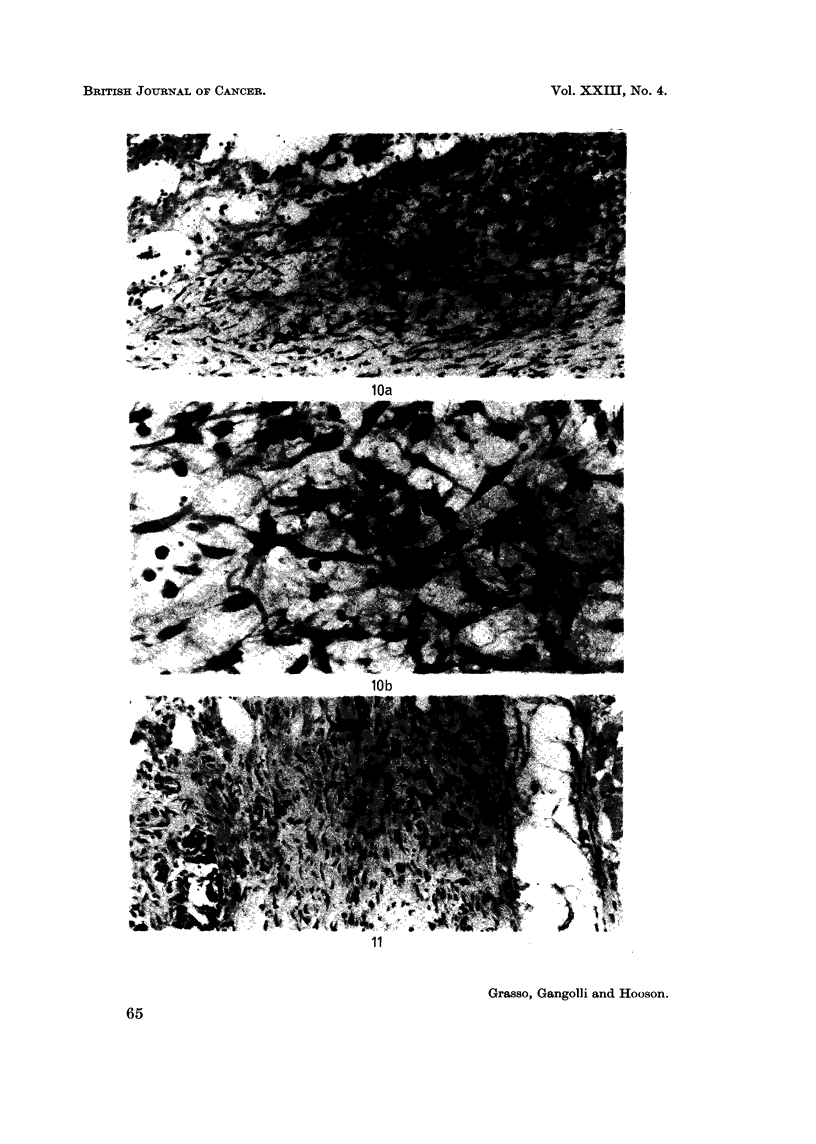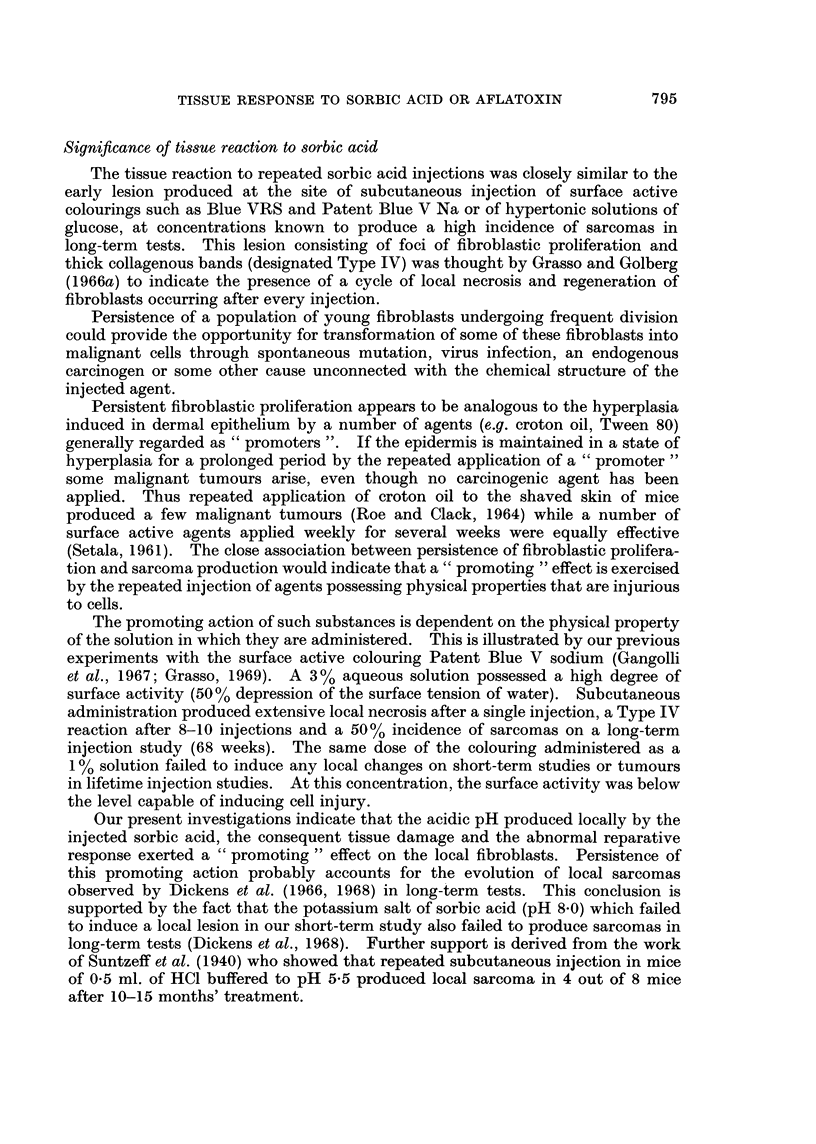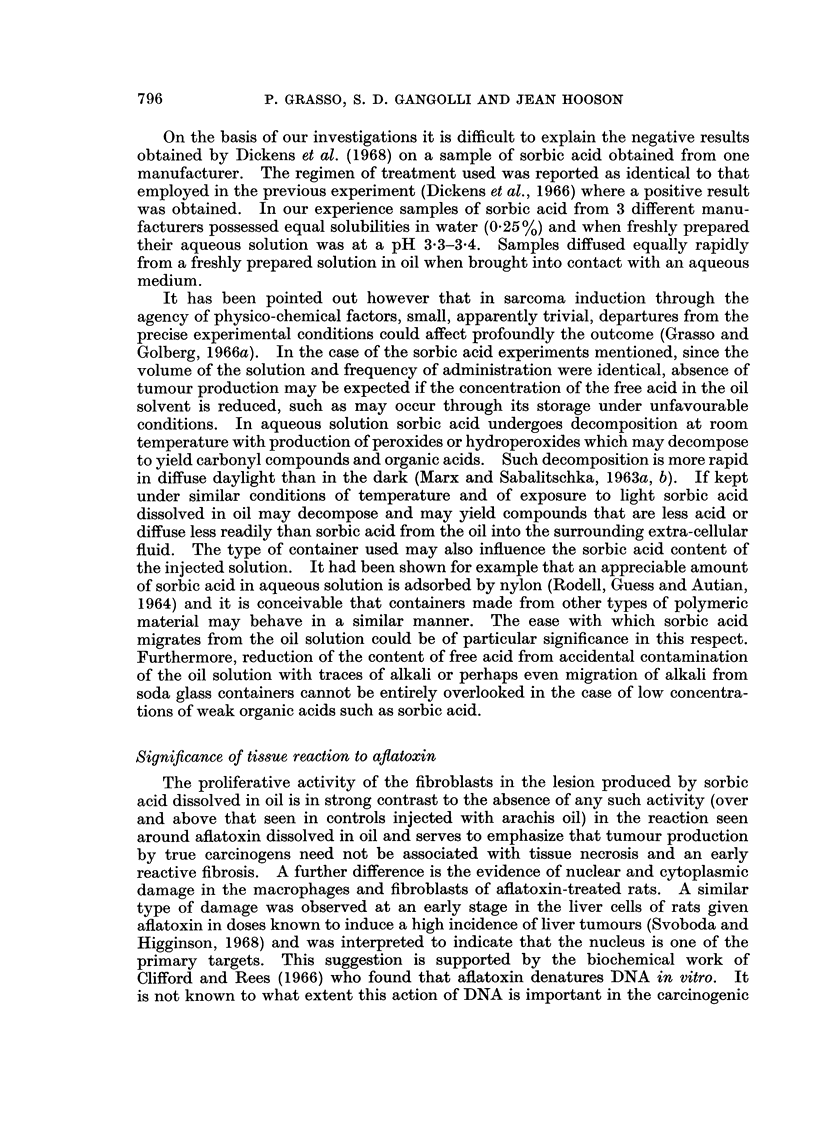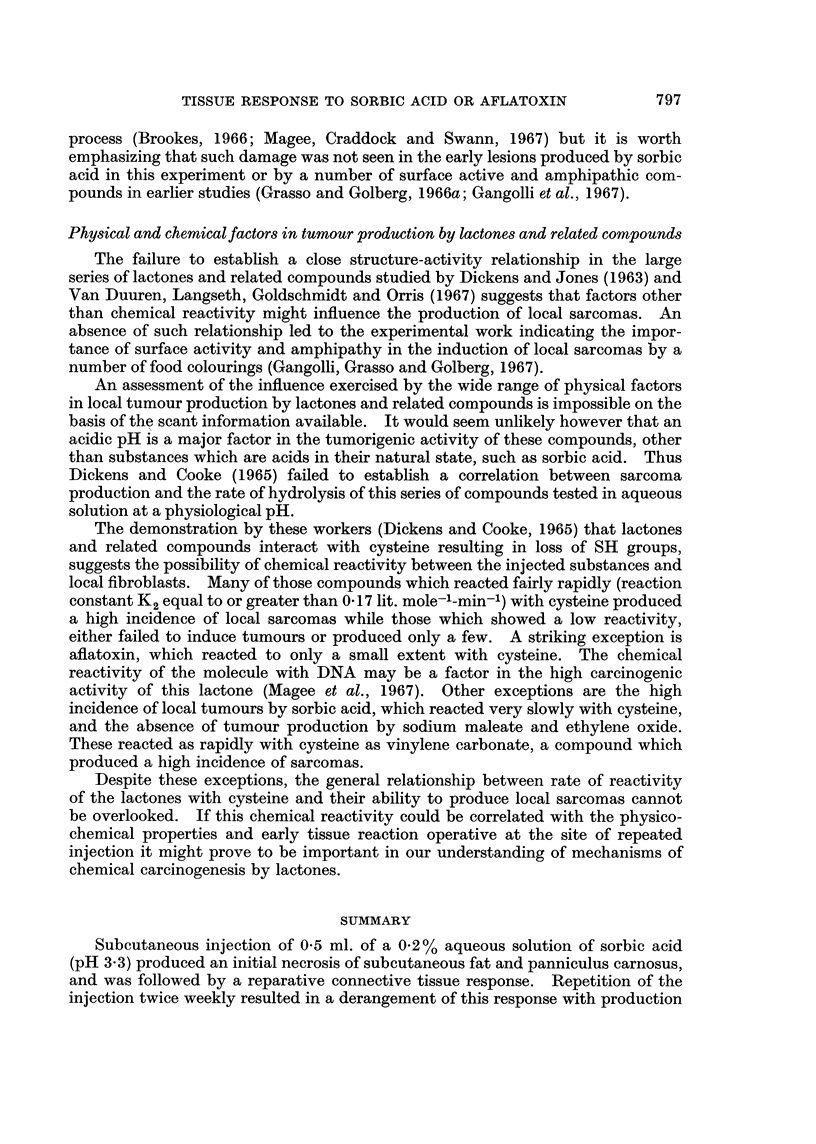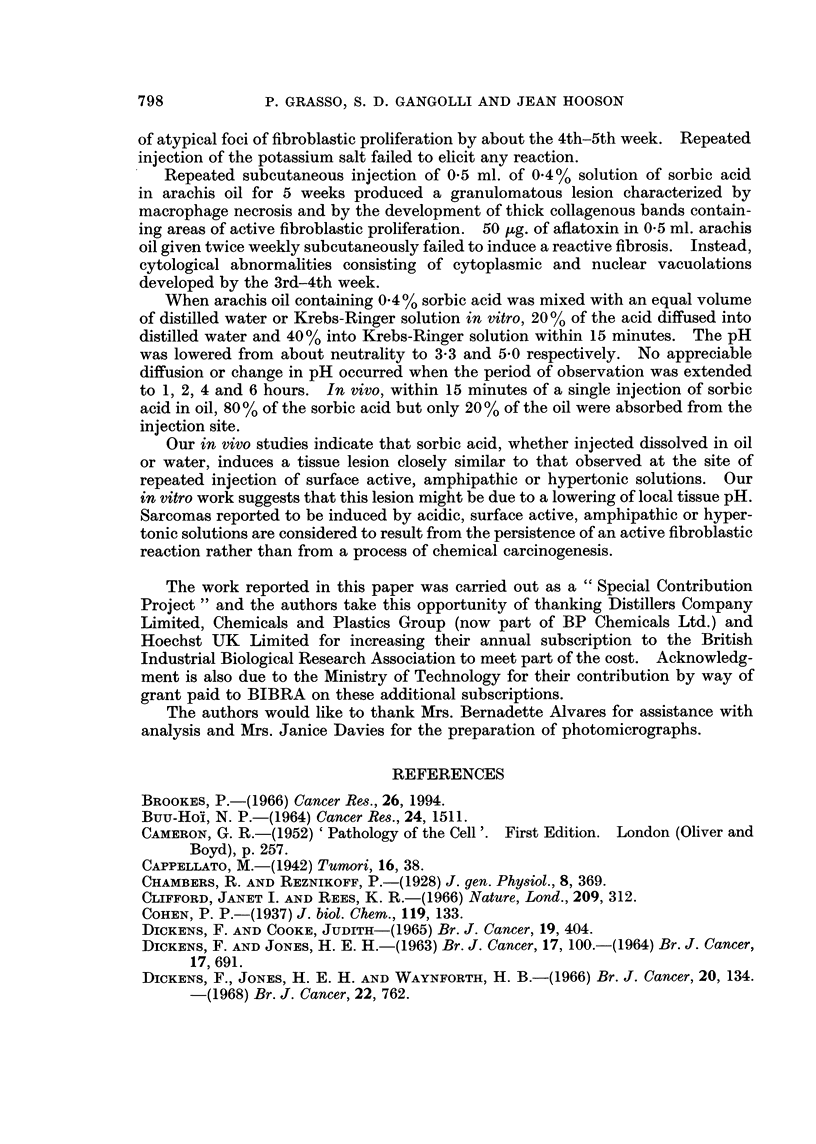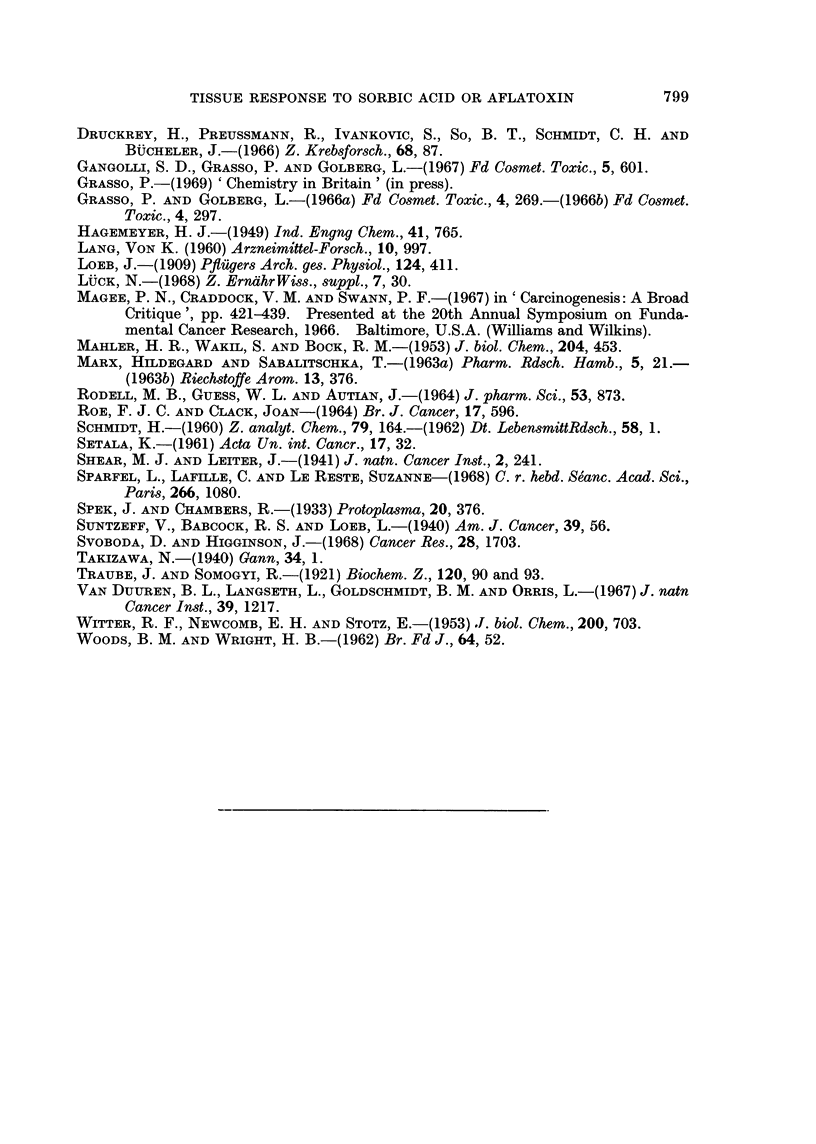# Connective tissue response to a short-term series of subcutaneous injections of sorbic acid or aflatoxin. Physico-chemical factors determining reaction to sorbic acid.

**DOI:** 10.1038/bjc.1969.98

**Published:** 1969-12

**Authors:** P. Grasso, S. D. Gangolli, J. Hooson

## Abstract

**Images:**


### CONNECTIVE TISSUE RESPONSE TO A SHORT-TERM SERIES OF SUBCUTANEOUS INJECTIONS OF SORBIC ACID OR AFLATOXIN. PHYSICO-CHEMICAL FACTORS DETERMINING REACTION TO SORBIC ACID

P. GRASSO, S. D. GANGOLLI AND JEAN HOOSON

*From the British Industrial Biological Research Association, Woodmansterne Road, Carshalton, Surrey*

Received for publication June 12, 1969

THE undoubted value of sorbic acid (Fig. 1) as a preservative in foodstuffs that are susceptible to spoilage by micro-organisms (Woods and Wright, 1962; Lück, 1968) has stimulated considerable interest in its safety under conditions of use and especially in any carcinogenic hazard it might present.

Concern about this hazard arose because sorbic acid was at first derived by acid hydrolysis of the lactone parasorbic acid (Fig. 1) found naturally in mountain-ash berries *Sorbus aucuparia L.* Parasorbic acid induced local sarcomas in 8 out of 11 rats after its repeated subcutaneous injection in doses of 0·2–2·0 mg., a finding which was consistent with similar observations on the carcinogenicity of other $\alpha$-, $\beta$-unsaturated lactones (Dickens and Jones, 1963). Production of sorbic acid from parasorbic acid is now chiefly of historical interest since modern methods of production of sorbic acid on a large scale for commercial purposes involve catalytic condensation of croton aldehyde and ketene (Hagemeyer, 1949).

Acute studies revealed that rats tolerated high doses of sorbic acid ($LD_{50}$ value 7·4 g./kg.) and chronic feeding tests over 2 years with 5% sorbic acid in the diet produced no ill effect on rats (Lang, 1960; Sparfel, Lafille and Le Reste, 1968). Metabolic studies showed that sorbic acid is qualitatively metabolized in the same manner as the saturated or singly unsaturated fatty acids of the same C-atom number and is readily utilized by the organism as a source of calories (Cohen, 1937; Witter, Newcomb and Stotz, 1953; Mahler, Wakil and Bock, 1953).

Although sorbic acid did not increase the natural incidence of tumours in rats when given in the diet (Lang, 1960), twice weekly subcutaneous injection for 65 weeks in rats of 2 mg. of the acid, obtained from one supplier and dissolved in 0·5 ml. arachis oil, resulted in the production of local sarcomas in 5 out of 6 treated rats (Dickens, Jones and Waynforth, 1966). In subsequent experiments using sorbic acid from another supplier, the result was negative (Dickens, Jones and Waynforth, 1968). A 0·4% aqueous solution of the acid obtained from the first supplier produced sarcomas in 2 out of 6 male rats treated with 0·5 ml. of the solution twice weekly subcutaneously for 56–60 weeks, while the potassium salt administered by the subcutaneous route in the same dosage and frequency and for the same period did not give rise to any tumours (Dickens *et al.*, 1968).

Sarcomas arise at the site of injection of a wide variety of known carcinogenic substances such as polycyclic aromatic hydrocarbons (Shear and Leiter, 1941;

Buu-Hoï, 1964), aflatoxin (Dickens and Jones, 1964) and a number of analogues of nitrosourea (Druckrey, Preussmann, Ivankovic, So, Schmidt and Bücheler, 1966).

In a review of the problem of subcutaneous sarcomas as an index of carcinogenic activity, Grasso and Golberg (1966*b*) drew attention to the fact that the production of locally malignant tumours does not necessarily indicate that the substance injected is carcinogenic. Repeated long-term injection of hydrochloric acid buffered at pH 5·0 (Suntzeff, Babcock and Loeb, 1940) and of hypertonic solutions of glucose and other monosaccharides (Takizawa, 1940) have been shown to produce a high incidence of local sarcomata.

A salient feature of the local reaction produced by a limited number of injections of hypertonic glucose solution is persistent fibroblastic proliferation accompanied by wide zones of necrosis and collagenization (Takizawa, 1940; Cappellato, 1942). A similar type of persistently active lesion (designated Type IV) was found to develop at the site of repeated subcutaneous injection of surface active and amphipathic (readily soluble in lipids and water) compounds (Grasso and Golberg, 1966*a*; Gangolli, Grasso and Golberg, 1967). These authors interpreted this persistently active lesion as indicating that repeated injections of surface active, amphipathic or hypertonic solutions prevented the process of connective tissue repair from producing a mature scar tissue, its natural endpoint, and that this block or inhibition of the reparative process was an essential factor in the emergence of local malignancy. Compounds that were structurally closely related to the surface active or amphipathic compounds studied, but were devoid of these physical properties, failed to induce a persistent type of lesion on short-term administration or to develop sarcoma on long-term injection tests.

These findings suggested the possibility that physical factors capable of inducing local tissue injury may be responsible for the malignant outcome at the site of repeated subcutaneous injection of sorbic acid.

We have investigated the tissue reaction produced by a short-term series of injections of sorbic acid dissolved either in oil or in water and compared them with the local reaction produced by the potent carcinogen aflatoxin. The lesion produced by sorbic acid dissolved in either medium was found to have a number of important features seen also in the reactive lesion produced by compounds exhibiting surface active, amphipathic or other physical properties (Gangolli *et al.*, 1967) but was different from the lesion produced by aflatoxin.

*In vitro* studies of the physico-chemical properties demonstrate that sorbic acid diffuses readily from the oil into the aqueous phase with consequent lowering of pH. On subcutaneous injection, sorbic acid diffuses rapidly from the oil solvent into the surrounding extracellular fluid and probably leads to a fall in pH with consequent cell damage and production of an atypical connective tissue response.

### MATERIALS AND METHODS

### *Rats*

Rats of the CFE strain of both sexes (wt 120–200 g.) were used throughout these experiments. They were fed on Spillers' Laboratory Small Animal Diet and were allowed free access to food and water. The animals, in groups of 4, were housed in metal cages with a grid floor. They were maintained at a temperature of 22 $\pm$ 1° C. and at 50% relative humidity.

*Chemicals*

Sorbic acid was dissolved to form a 0·4% solution in arachis oil and a 0·2% solution in distilled water. Potassium sorbate was dissolved in water to form a 0·2% solution. These solutions were freshly prepared each week. Both compounds were obtained from Pfizer Ltd., Sandwich, Kent.

Aflatoxin mixture B and G (Fig. 1) was obtained from the Medical Research Council. The analytical data (W. Lijinsky, 1965, personal communication) are

Fig. 1.—Structural formula of sorbic acid, parasorbic acid and aflatoxin $B_1$ and $G_1$.

as follows: $B_1$ 25%; $B_2$ 14·6%; $G_1$ 50·7%; $G_2$ 7·15%. The mixture was dissolved in arachis oil in a concentration of 100 $\mu$g./ml. Arachis oil was obtained from British Drug Houses Ltd., Poole, Dorset. Glyceryl tri(stearate-1-$^{14}$C) was obtained from the Radiochemical Centre, Amersham.

*Absorption studies* in vivo

The rates of absorption of sorbic acid and its oil solvent were estimated from the difference between the amount injected and the amount of oil or sorbic acid remaining at the site after stated intervals of time. A single subcutaneous injection of 0·5 ml. of a 0·4% solution of sorbic acid in arachis oil containing $^{14}$C labelled glyceryl tristearate (100,000 c.p.m. per 0·5 ml.) was administered to a group of 48 rats. Oil Red O was added to the oil as a visual marker to assist sampling the injection site at necropsy.

Groups of 6 rats were killed at 0, 15 minutes, 1, 2, 4, 8 and 24 hours, and the subcutaneous site excised according to the method used by Gangolli *et al.* (1967). Three rats from each group were used to study the rate of absorption of the acid, and the remaining 3 for the rate of absorption of the oil.

For the determination of the amount of sorbic acid at the site of injections the tissue was ground with anhydrous sodium sulphate AR and extracted with 50 ml. of methanol AR in a soxhlet extractor for 4 hours. The methanolic extracts were cooled and diluted to 100 ml. 18 ml. of this solution was made up to 20 ml. with 0·2N $H_2SO_4$ and filtered. One drop of 5N alcoholic KOH was added to 2 ml. of the filtrate which was then evaporated to dryness. The residue was dissolved in 10 ml. distilled water and the sorbic acid determined by the colorimetric method of Schmidt (1960, 1962).

The local residue of labelled stearate in the oil was determined on the tissues removed from the remainder of the groups by grinding with anhydrous sodium sulphate AR and extraction of the fat with diethyl ether AR in a soxhlet extractor for 4 hours. The ether extracts were evaporated to dryness and the fatty residues dissolved in 1·0 ml. of toluene AR. 0·1 ml. of this toluene solution was diluted with scintillating fluid (PPO-dimethyl POPOP) and counted in a Nuclear-Chicago 725 scintillation counter.

*Diffusion studies* in vitro

The degree of diffusion of sorbic acid from the oil phase into an aqueous phase consisting of either $CO_2$-free distilled water or Krebs-Ringer buffer (pH 7·4) was investigated. 50 ml. of 0·4% solution of sorbic acid in oil were mixed with an equal volume of distilled water or Krebs-Ringer buffer. The mixture was kept at room temperature (22° C.) and stirred vigorously. Measurement of the pH of the aqueous phase was taken at intervals of 1 minute for the first 15 minutes and subsequently at 1, 2, 3 and 4 hours.

The amount of sorbic acid in the respective aqueous phase was determined at 15 minutes, 1, 2, 3 and 4 hours by the u.v. absorption at 258 m$\mu$.

*Study of tissue reaction*

Groups of 20 rats (10 M and 10 F) were given twice weekly subcutaneous injections of 0·5 ml. arachis oil alone or containing 2 mg. of sorbic acid or 50 $\mu$g. aflatoxin. Groups of the same number of rats were given 0·5 ml. of an aqueous solution of 0·2% sorbic acid or potassium sorbate subcutaneously twice weekly. Injections were given at the same site as far as possible.

From each of these 5 groups 4 animals were killed each week, and the injection site excised, fixed and prepared for histological examination as described by Grasso and Golberg (1966*a*). Experiments were terminated at the end of the 5th week. For electron microscopy the injection site was excised, cut into 3–5 mm. cubes of subcutaneous tissue and overlying skin and fixed in cacodylate buffered osmium tetroxide for 1 hour. Rapid dehydration was followed by embedding in Epon 812. 2–3 $\mu$ sections were cut and stained with 1% toluidine blue in 1% borax. The relevant cells were identified by light microscopy, and ultrathin (800 Å–1000 Å) sections of selected areas cut. Sections were mounted on Formvar grids, stained with uranyl acetate followed by lead citrate and examined with an AEI-EM6B electron microscope.

### RESULTS

*Absorption studies* in vivo

Rates of absorption of sorbic acid and of the oil from the site of subcutaneous injection of a 0·4% solution of sorbic acid in oil are given in Fig. 2. 80% of the injected dose of sorbic acid was absorbed by 15 minutes, whereas only 5% of the oil was lost at this time. Subsequently the rate of absorption of the remainder

FIG. 2.—Absorption of sorbic acid and arachis oil from the site of a single subcutaneous injection of 0·4% sorbic acid in arachis oil containing glyceryl tri(stearate-1-$^{14}$C).

of the sorbic acid and the oil was roughly similar. At the end of the 24 hour period, no sorbic acid was found at the injection site but 25% of the amount of oil injected was recovered.

*Diffusion studies* in vitro

The amounts of sorbic acid that diffused out of the oil phase into an aqueous phase consisting of distilled water or Krebs-Ringer are given in Fig. 3. Within

FIG. 3.—Diffusion of sorbic acid from a 0·4% solution in oil into an equal volume of Krebs-Ringer solution or distilled water.

15 minutes, 35% of the sorbic acid originally contained in the oil phase was found in Krebs-Ringer and 18% in distilled water. At the end of 4 hours, 50% of the sorbic acid had diffused into Krebs-Ringer and 25% into distilled water.

Measurement of pH during the first 15 minutes of contact between distilled water and the oil phase revealed that a pH of 3·3 in the aqueous phase was achieved within 1 minute. The pH change using Krebs-Ringer buffered solution was more gradual but a maximum drop to pH 5 was reached by 15 minutes (Fig. 4). No further change in pH was observed when measurements were taken at 1, 2, 3 and 4 hours.

FIG. 4.—Change in the pH of distilled water or Krebs-Ringer solution mixed with an equal volume of 0·4% sorbic acid in oil.

*Tissue reactions to arachis oil, sorbic acid or aflatoxin in oil*

Injection of arachis oil produced a granulomatous reaction consisting of numerous oil deposits surrounded by a single or double layer of macrophages often accompanied by a mononuclear and round cell infiltration. From the 3rd injection until the end of the experiment (5th week), flattened endothelial-like cells and a few thin strands of fibrous tissue were seen around the oil globules (Fig. 5).

The reactions observed after each of the first 3 injections of aflatoxin in oil consisted of an infiltration by mononuclear and lymphocytic cells which formed dense zones around some of the oil deposits. A layer of macrophages was seen to surround the oil droplets from the 3rd injection onwards but this layer often failed to isolate completely the oil from the subcutaneous tissue. In some instances the oil droplets were surrounded partially or completely by a layer of fibrin. In the later stages (7th–10th injections), atypical fibroblasts and macrophages containing vacuolated cytoplasm, an increased amount of RNA and vesicular nuclei containing large nucleoli were present in the granulomatous reaction, and abnormal mitoses were detected (Fig. 6a, b and c). The nuclear abnormalities were confirmed ultrastructurally (Fig. 6d).

The oil residue in rats killed after the 1st injection of sorbic acid in oil was substantially less than that observed on injecting the oil alone or oil containing aflatoxin. Histologically the subcutaneous site contained considerable fibrin deposits and necrotic fat cells. Subsequently (3–6 injections) oil deposits accumulated subcutaneously and were histologically surrounded by fibrin and macrophages, many of which were necrotic. A marked fibroblastic reaction accompanied these changes which, with the progress of the experiment was accompanied by the formation of thick connective tissue bands, rich in hyaline collagen, in between the oil droplets (Fig. 7). In animals that received 8 or more injections, foci of fibroblastic proliferation could occasionally be found (Fig. 8). A summary of these findings is given in Table I. The thick connective tissue seen histologically accounted for the progressive thickening and hardening observed at the injection site during the experiment.

TABLE I.—*Local Connective Tissue Response (A–C) to Repeated Injection of Sorbic Acid Dissolved in Oil*

| Number of injections | Incidence of connective tissue response* | | |
|---|---|---|---|
| | A | B | C |
| 1–2 | 3/4† | — | — |
| 3–4 | 3/4 | 3/4 | — |
| 5–10 | — | 9/12 | 2/12 |

* A = Tissue necrosis followed by development of oil granuloma.
B = Thick connective tissue bands separating oil deposits. Necrosis of macrophages or fibroblasts involving single or groups of cells present.
C = Foci of fibroblastic proliferation.
† Values cited are the number of rats in which observation was made out of the total number of rats examined.

*Studies of the tissue reactions to aqueous solutions of sorbic acid and potassium sorbate*

The first injection of sorbic acid produced extensive necrosis of the subcutaneous fat and panniculus muscle. Fibrin deposits accompanied by proliferation of connective tissue cells replaced the necrotic tissue and were the principal pathological features of the lesion seen after the 1st or 2nd injection. After the 3rd injection granulation tissue filled the injection site (Fig. 9). Necrosis of fibroblasts was seen after the 4th and subsequent injections. In addition, the granulation tissue did not progress to form a mature scar tissue. Instead there was fibroblastic proliferation forming atypical zones or foci, which persisted until the end of the experiment (10th week) (Fig. 10a, b). Extensive deposition of collagen was often found at this stage (Fig. 11). These findings are summarized in Table II.

Potassium sorbate failed to elicit a connective tissue response.

TABLE II.—*Local Connective Tissue Response to Repeated Injection of Sorbic Acid Dissolved in Water*

| Number of injections | Incidence of connective tissue response* | | |
|---|---|---|---|
| | A | B | C |
| 1–2 | 4/4† | — | — |
| 3–4 | 4/4 | 1/4 | — |
| 5–10 | 2/12 | 5/12 | 2/12 |

* A = Necrosis of subcutaneous tissue followed by inflammation and a reparative response.
B = Formation of connective tissue with thick collagenous bands.
C = Foci of proliferating fibroblasts in a state of disarray.
† Values cited are the number of rats in which observation was made out of the total number of rats examined.

### DISCUSSION

*Lowering of pH and cell damage*

Sorbic acid is appreciably soluble in water (0·25%) and the resulting solution is considerably more acid (pH 3·3) than body fluids (generally regarded as pH 7·4).

The readiness with which sorbic acid diffuses from the oil phase into distilled water and the even more complete diffusion into Krebs-Ringer solution may account for the rapid absorption of sorbic acid compared with the oily vehicle from the site of injection. The change in pH of these aqueous media as a result of diffusion of sorbic acid suggests that its injection as a solution in oil may expose the tissue to the effects of a change in pH from physiological to acid conditions almost as effectively as the injection of an aqueous solution of sorbic acid.

Studies on unicellular organisms and on cells in tissue culture have shown that lowering the pH to 6·0 decreases movement and multiplication (Cameron, 1952). At lower pH levels damage becomes pronounced and irreversible (Spek and Chambers, 1933). Organic acids were found by Loeb (1909) to be more toxic than mineral acids and he explained this difference by the former's greater solubility in lipids.

The mode of action of acids on the cell is a complicated one, and though the toxic effect is usually attributed to hydrogen ion concentration there is no complete parallelism between toxic action and pH (Cameron, 1952). According to Traube and Somogyi (1921) acids are " capillary active " and become concentrated at the cell surface where they exert their " poisonous " effects. In support of this suggestion Chambers and Reznikoff (1928) demonstrated that at pH 5·5 HCl is toxic to the cell surface whereas introduction of acid pH 2·5 inside the cell produced a reversible injury because of the efficient buffering systems in the cytoplasm.

On the basis of these experimental findings the tissue necrosis and the development of an abnormal type of tissue reaction found in our studies are consistent with a local lowering of tissue pH at the site of repeated injection of sorbic acid in oil or water. The absence of tissue injury when injecting K-sorbate supports this suggestion.

---

### EXPLANATION OF PLATES

FIG. 5.—Granuloma at site of 10 injections of 0·5 ml. of arachis oil. H. and E. × 30.

FIG. 6a.—Connective tissue septum containing macrophages and fibroblasts with prominent cytoplasmic and nuclear vacuolations. Rat injected with 50 μg. aflatoxin in 0·5 ml. arachis oil twice weekly for 3 weeks. H. and E. × 30.

FIG. 6b.—Vacuolated macrophages. H. and E. × 750.

FIG. 6c.—Vacuolated fibroblast. H. and E. × 750.

FIG. 6d.—Electron micrograph of vacuolated macrophage, showing margination and condensation of chromatin. × 11,250.

FIG. 7.—Large oil deposits surrounded by thick connective tissue septa in the subcutaneous tissue of the rat treated with 6 injections of 0·4% sorbic acid in arachis oil. H. and E. × 30.

FIG. 8.—Proliferating fibroblasts in thick connective tissue septum separating oil deposits. Rat given 8 subcutaneous injections of 0·5 ml. of 0 4% sorbic acid in oil. H. and E. × 200.

FIG. 9.—Granulation tissue at the site of 3 subcutaneous injections of 0·5 ml. of 0·2% aqueous solution of sorbic acid. H. and E. × 200.

FIG. 10a.—Atypical proliferation of fibroblasts at the site of 9 subcutaneous injections of 0·5 ml. of 0·2% aqueous solution of sorbic acid. H. and E. × 28.

FIG. 10b.—Higher magnification of above. H. and E. × 280.

FIG. 11.—Thick connective tissue containing young fibroblasts in the subcutaneous tissue of a rat treated with 8 injections of 0·5 ml. of 0·2% aqueous solution of sorbic acid. H. and E. × 175.

5

6a

6b

6c

Grasso, Gangolli and Hooson.

6d

7

8

9

Grasso, Gangolli and Hooson.

10a

10b

11

Grasso, Gangolli and Hooson.

*Significance of tissue reaction to sorbic acid*

The tissue reaction to repeated sorbic acid injections was closely similar to the early lesion produced at the site of subcutaneous injection of surface active colourings such as Blue VRS and Patent Blue V Na or of hypertonic solutions of glucose, at concentrations known to produce a high incidence of sarcomas in long-term tests. This lesion consisting of foci of fibroblastic proliferation and thick collagenous bands (designated Type IV) was thought by Grasso and Golberg (1966*a*) to indicate the presence of a cycle of local necrosis and regeneration of fibroblasts occurring after every injection.

Persistence of a population of young fibroblasts undergoing frequent division could provide the opportunity for transformation of some of these fibroblasts into malignant cells through spontaneous mutation, virus infection, an endogenous carcinogen or some other cause unconnected with the chemical structure of the injected agent.

Persistent fibroblastic proliferation appears to be analogous to the hyperplasia induced in dermal epithelium by a number of agents (*e.g.* croton oil, Tween 80) generally regarded as " promoters ". If the epidermis is maintained in a state of hyperplasia for a prolonged period by the repeated application of a " promoter " some malignant tumours arise, even though no carcinogenic agent has been applied. Thus repeated application of croton oil to the shaved skin of mice produced a few malignant tumours (Roe and Clack, 1964) while a number of surface active agents applied weekly for several weeks were equally effective (Setala, 1961). The close association between persistence of fibroblastic proliferation and sarcoma production would indicate that a " promoting " effect is exercised by the repeated injection of agents possessing physical properties that are injurious to cells.

The promoting action of such substances is dependent on the physical property of the solution in which they are administered. This is illustrated by our previous experiments with the surface active colouring Patent Blue V sodium (Gangolli *et al.*, 1967; Grasso, 1969). A 3% aqueous solution possessed a high degree of surface activity (50% depression of the surface tension of water). Subcutaneous administration produced extensive local necrosis after a single injection, a Type IV reaction after 8–10 injections and a 50% incidence of sarcomas on a long-term injection study (68 weeks). The same dose of the colouring administered as a 1% solution failed to induce any local changes on short-term studies or tumours in lifetime injection studies. At this concentration, the surface activity was below the level capable of inducing cell injury.

Our present investigations indicate that the acidic pH produced locally by the injected sorbic acid, the consequent tissue damage and the abnormal reparative response exerted a " promoting " effect on the local fibroblasts. Persistence of this promoting action probably accounts for the evolution of local sarcomas observed by Dickens *et al.* (1966, 1968) in long-term tests. This conclusion is supported by the fact that the potassium salt of sorbic acid (pH 8·0) which failed to induce a local lesion in our short-term study also failed to produce sarcomas in long-term tests (Dickens *et al.*, 1968). Further support is derived from the work of Suntzeff *et al.* (1940) who showed that repeated subcutaneous injection in mice of 0·5 ml. of HCl buffered to pH 5·5 produced local sarcoma in 4 out of 8 mice after 10–15 months' treatment.

On the basis of our investigations it is difficult to explain the negative results obtained by Dickens *et al.* (1968) on a sample of sorbic acid obtained from one manufacturer. The regimen of treatment used was reported as identical to that employed in the previous experiment (Dickens *et al.*, 1966) where a positive result was obtained. In our experience samples of sorbic acid from 3 different manufacturers possessed equal solubilities in water (0·25%) and when freshly prepared their aqueous solution was at a pH 3·3–3·4. Samples diffused equally rapidly from a freshly prepared solution in oil when brought into contact with an aqueous medium.

It has been pointed out however that in sarcoma induction through the agency of physico-chemical factors, small, apparently trivial, departures from the precise experimental conditions could affect profoundly the outcome (Grasso and Golberg, 1966*a*). In the case of the sorbic acid experiments mentioned, since the volume of the solution and frequency of administration were identical, absence of tumour production may be expected if the concentration of the free acid in the oil solvent is reduced, such as may occur through its storage under unfavourable conditions. In aqueous solution sorbic acid undergoes decomposition at room temperature with production of peroxides or hydroperoxides which may decompose to yield carbonyl compounds and organic acids. Such decomposition is more rapid in diffuse daylight than in the dark (Marx and Sabalitschka, 1963*a*, *b*). If kept under similar conditions of temperature and of exposure to light sorbic acid dissolved in oil may decompose and may yield compounds that are less acid or diffuse less readily than sorbic acid from the oil into the surrounding extra-cellular fluid. The type of container used may also influence the sorbic acid content of the injected solution. It had been shown for example that an appreciable amount of sorbic acid in aqueous solution is adsorbed by nylon (Rodell, Guess and Autian, 1964) and it is conceivable that containers made from other types of polymeric material may behave in a similar manner. The ease with which sorbic acid migrates from the oil solution could be of particular significance in this respect. Furthermore, reduction of the content of free acid from accidental contamination of the oil solution with traces of alkali or perhaps even migration of alkali from soda glass containers cannot be entirely overlooked in the case of low concentrations of weak organic acids such as sorbic acid.

*Significance of tissue reaction to aflatoxin*

The proliferative activity of the fibroblasts in the lesion produced by sorbic acid dissolved in oil is in strong contrast to the absence of any such activity (over and above that seen in controls injected with arachis oil) in the reaction seen around aflatoxin dissolved in oil and serves to emphasize that tumour production by true carcinogens need not be associated with tissue necrosis and an early reactive fibrosis. A further difference is the evidence of nuclear and cytoplasmic damage in the macrophages and fibroblasts of aflatoxin-treated rats. A similar type of damage was observed at an early stage in the liver cells of rats given aflatoxin in doses known to induce a high incidence of liver tumours (Svoboda and Higginson, 1968) and was interpreted to indicate that the nucleus is one of the primary targets. This suggestion is supported by the biochemical work of Clifford and Rees (1966) who found that aflatoxin denatures DNA *in vitro*. It is not known to what extent this action of DNA is important in the carcinogenic process (Brookes, 1966; Magee, Craddock and Swann, 1967) but it is worth emphasizing that such damage was not seen in the early lesions produced by sorbic acid in this experiment or by a number of surface active and amphipathic compounds in earlier studies (Grasso and Golberg, 1966*a*; Gangolli *et al.*, 1967).

*Physical and chemical factors in tumour production by lactones and related compounds*

The failure to establish a close structure-activity relationship in the large series of lactones and related compounds studied by Dickens and Jones (1963) and Van Duuren, Langseth, Goldschmidt and Orris (1967) suggests that factors other than chemical reactivity might influence the production of local sarcomas. An absence of such relationship led to the experimental work indicating the importance of surface activity and amphipathy in the induction of local sarcomas by a number of food colourings (Gangolli, Grasso and Golberg, 1967).

An assessment of the influence exercised by the wide range of physical factors in local tumour production by lactones and related compounds is impossible on the basis of the scant information available. It would seem unlikely however that an acidic pH is a major factor in the tumorigenic activity of these compounds, other than substances which are acids in their natural state, such as sorbic acid. Thus Dickens and Cooke (1965) failed to establish a correlation between sarcoma production and the rate of hydrolysis of this series of compounds tested in aqueous solution at a physiological pH.

The demonstration by these workers (Dickens and Cooke, 1965) that lactones and related compounds interact with cysteine resulting in loss of SH groups, suggests the possibility of chemical reactivity between the injected substances and local fibroblasts. Many of those compounds which reacted fairly rapidly (reaction constant $K_2$ equal to or greater than 0·17 lit. mole$^{-1}$-min$^{-1}$) with cysteine produced a high incidence of local sarcomas while those which showed a low reactivity, either failed to induce tumours or produced only a few. A striking exception is aflatoxin, which reacted to only a small extent with cysteine. The chemical reactivity of the molecule with DNA may be a factor in the high carcinogenic activity of this lactone (Magee *et al.*, 1967). Other exceptions are the high incidence of local tumours by sorbic acid, which reacted very slowly with cysteine, and the absence of tumour production by sodium maleate and ethylene oxide. These reacted as rapidly with cysteine as vinylene carbonate, a compound which produced a high incidence of sarcomas.

Despite these exceptions, the general relationship between rate of reactivity of the lactones with cysteine and their ability to produce local sarcomas cannot be overlooked. If this chemical reactivity could be correlated with the physico-chemical properties and early tissue reaction operative at the site of repeated injection it might prove to be important in our understanding of mechanisms of chemical carcinogenesis by lactones.

### SUMMARY

Subcutaneous injection of 0·5 ml. of a 0·2% aqueous solution of sorbic acid (pH 3·3) produced an initial necrosis of subcutaneous fat and panniculus carnosus, and was followed by a reparative connective tissue response. Repetition of the injection twice weekly resulted in a derangement of this response with production of atypical foci of fibroblastic proliferation by about the 4th–5th week. Repeated injection of the potassium salt failed to elicit any reaction.

Repeated subcutaneous injection of 0·5 ml. of 0·4% solution of sorbic acid in arachis oil for 5 weeks produced a granulomatous lesion characterized by macrophage necrosis and by the development of thick collagenous bands containing areas of active fibroblastic proliferation. 50 $\mu$g. of aflatoxin in 0·5 ml. arachis oil given twice weekly subcutaneously failed to induce a reactive fibrosis. Instead, cytological abnormalities consisting of cytoplasmic and nuclear vacuolations developed by the 3rd–4th week.

When arachis oil containing 0·4% sorbic acid was mixed with an equal volume of distilled water or Krebs-Ringer solution *in vitro*, 20% of the acid diffused into distilled water and 40% into Krebs-Ringer solution within 15 minutes. The pH was lowered from about neutrality to 3·3 and 5·0 respectively. No appreciable diffusion or change in pH occurred when the period of observation was extended to 1, 2, 4 and 6 hours. *In vivo*, within 15 minutes of a single injection of sorbic acid in oil, 80% of the sorbic acid but only 20% of the oil were absorbed from the injection site.

Our *in vivo* studies indicate that sorbic acid, whether injected dissolved in oil or water, induces a tissue lesion closely similar to that observed at the site of repeated injection of surface active, amphipathic or hypertonic solutions. Our *in vitro* work suggests that this lesion might be due to a lowering of local tissue pH. Sarcomas reported to be induced by acidic, surface active, amphipathic or hypertonic solutions are considered to result from the persistence of an active fibroblastic reaction rather than from a process of chemical carcinogenesis.

The work reported in this paper was carried out as a " Special Contribution Project " and the authors take this opportunity of thanking Distillers Company Limited, Chemicals and Plastics Group (now part of BP Chemicals Ltd.) and Hoechst UK Limited for increasing their annual subscription to the British Industrial Biological Research Association to meet part of the cost. Acknowledgment is also due to the Ministry of Technology for their contribution by way of grant paid to BIBRA on these additional subscriptions.

The authors would like to thank Mrs. Bernadette Alvares for assistance with analysis and Mrs. Janice Davies for the preparation of photomicrographs.

### REFERENCES

Brookes, P.—(1966) *Cancer Res.*, **26**, 1994.

Buu-Hoï, N. P.—(1964) *Cancer Res.*, **24**, 1511.

Cameron, G. R.—(1952) ' Pathology of the Cell '. First Edition. London (Oliver and Boyd), p. 257.

Cappellato, M.—(1942) *Tumori*, **16**, 38.

Chambers, R. and Reznikoff, P.—(1928) *J. gen. Physiol.*, **8**, 369.

Clifford, Janet I. and Rees, K. R.—(1966) *Nature, Lond.*, **209**, 312.

Cohen, P. P.—(1937) *J. biol. Chem.*, **119**, 133.

Dickens, F. and Cooke, Judith—(1965) *Br. J. Cancer*, **19**, 404.

Dickens, F. and Jones, H. E. H.—(1963) *Br. J. Cancer*, **17**, 100.—(1964) *Br. J. Cancer*, **17**, 691.

Dickens, F., Jones, H. E. H. and Waynforth, H. B.—(1966) *Br. J. Cancer*, **20**, 134. —(1968) *Br. J. Cancer*, **22**, 762.

DRUCKREY, H., PREUSSMANN, R., IVANKOVIC, S., SO, B. T., SCHMIDT, C. H. AND BÜCHELER, J.—(1966) *Z. Krebsforsch.*, **68**, 87.
GANGOLLI, S. D., GRASSO, P. AND GOLBERG, L.—(1967) *Fd Cosmet. Toxic.*, **5**, 601.
GRASSO, P.—(1969) ' Chemistry in Britain ' (in press).
GRASSO, P. AND GOLBERG, L.—(1966*a*) *Fd Cosmet. Toxic.*, **4**, 269.—(1966*b*) *Fd Cosmet. Toxic.*, **4**, 297.
HAGEMEYER, H. J.—(1949) *Ind. Engng Chem.*, **41**, 765.
LANG, VON K. (1960) *Arzneimittel-Forsch.*, **10**, 997.
LOEB, J.—(1909) *Pflügers Arch. ges. Physiol.*, **124**, 411.
LÜCK, N.—(1968) *Z. ErnährWiss.*, *suppl.*, **7**, 30.
MAGEE, P. N., CRADDOCK, V. M. AND SWANN, P. F.—(1967) in ' Carcinogenesis: A Broad Critique ', pp. 421–439. Presented at the 20th Annual Symposium on Fundamental Cancer Research, 1966. Baltimore, U.S.A. (Williams and Wilkins).
MAHLER, H. R., WAKIL, S. AND BOCK, R. M.—(1953) *J. biol. Chem.*, **204**, 453.
MARX, HILDEGARD AND SABALITSCHKA, T.—(1963*a*) *Pharm. Rdsch. Hamb.*, **5**, 21.—(1963*b*) *Riechstoffe Arom.* **13**, 376.
RODELL, M. B., GUESS, W. L. AND AUTIAN, J.—(1964) *J. pharm. Sci.*, **53**, 873.
ROE, F. J. C. AND CLACK, JOAN—(1964) *Br. J. Cancer*, **17**, 596.
SCHMIDT, H.—(1960) *Z. analyt. Chem.*, **79**, 164.—(1962) *Dt. LebensmittRdsch.*, **58**, 1.
SETALA, K.—(1961) *Acta Un. int. Cancr.*, **17**, 32.
SHEAR, M. J. AND LEITER, J.—(1941) *J. natn. Cancer Inst.*, **2**, 241.
SPARFEL, L., LAFILLE, C. AND LE RESTE, SUZANNE—(1968) *C. r. hebd. Séanc. Acad. Sci., Paris*, **266**, 1080.
SPEK, J. AND CHAMBERS, R.—(1933) *Protoplasma*, **20**, 376.
SUNTZEFF, V., BABCOCK, R. S. AND LOEB, L.—(1940) *Am. J. Cancer*, **39**, 56.
SVOBODA, D. AND HIGGINSON, J.—(1968) *Cancer Res.*, **28**, 1703.
TAKIZAWA, N.—(1940) *Gann*, **34**, 1.
TRAUBE, J. AND SOMOGYI, R.—(1921) *Biochem. Z.*, **120**, 90 and 93.
VAN DUUREN, B. L., LANGSETH, L., GOLDSCHMIDT, B. M. AND ORRIS, L.—(1967) *J. natn Cancer Inst.*, **39**, 1217.
WITTER, R. F., NEWCOMB, E. H. AND STOTZ, E.—(1953) *J. biol. Chem.*, **200**, 703.
WOODS, B. M. AND WRIGHT, H. B.—(1962) *Br. Fd J.*, **64**, 52.